# A rapid increase in tropical species of grouper (Perciformes: Serranidae) in the temperate waters, the Goto Islands, Japan

**DOI:** 10.1371/journal.pone.0308715

**Published:** 2024-09-18

**Authors:** Junichi Okuyama, Masahiro Nakagawa, Takeshi Taneda

**Affiliations:** 1 Fisheries Technology Institute, Japan Fisheries Research and Education Agency, Ishigaki, Okinawa, Japan; 2 Fisheries Technology Institute, Japan Fisheries Research and Education Agency, Shibushi, Kagoshima, Japan; 3 Fisheries Resource Institute, Japan Fisheries Research and Education Agency, Nagasaki, Nagasaki, Japan; National Cheng Kung University, TAIWAN

## Abstract

Global warming has resulted in rapid poleward shifts in the geographical distributions of many tropical fish species. This study conducted daily market surveys from 2008 to 2013 to investigate catch trends of seven commercially important grouper species in the temperate Goto Islands, Japan. Our results revealed that the catch numbers of tropical grouper species increased rapidly by an average of 5.9-fold (12.3-fold at maximum) within six years, whereas the temperate and subtropical species did not exhibit substantial changes. Based on the findings of several previous studies, the rapid increase in the number of tropical groupers in temperate waters was most likely caused by the successful settlement of larvae transported from tropical waters. Large-scale ocean currents may facilitate larval transport from tropical waters because the Goto Islands face the Tsushima Warm Current, which branches from the Kuroshio Current. Meanwhile, the transition processes of size distribution in tropical groupers suggest a possible hypothesis that adults migrating from tropical waters first settle in temperate waters and then enhance their populations by reproduction. Further studies are required to determine how tropical grouper species settle and how their populations increase in temperate waters.

## Introduction

Global warming has resulted in rapid poleward shifts in the geographical distribution of many tropical fish species [1–10], affecting the composition of fish species in commercial fisheries [5, 11]. To predict the impact of global warming on ecosystems and commercial fisheries and to mitigate these impacts, it is essential to understand the mechanism of poleward shifts in the distribution of tropical fish [10]. Poleward shifts in the distribution of tropical fish are commonly considered to be associated with the successful settlement of tropical fish larvae into temperate waters [5, 7, 10]. Previously, when fish eggs, larvae, and juveniles were dispersed along currents and entered temperate waters from tropical waters, they did not survive because of physiological/ecological constraints, particularly at cold temperatures in winter [6, 8]. However, as a result of warming, they do not die, but successfully survive, settle, overwinter, and reproduce in temperate waters; their offspring subsequently contribute to the species’ population growth [1, 3, 4]. Although many studies have reported poleward shifts in the distribution of tropical fish, to the best of our knowledge, few have provided empirical evidence on how tropical fish increase in number and establish their populations in temperate waters, and the transition dynamics of populations during the expansion process (e.g., [1, 8]). To address this question, it is necessary to assess changes in the abundance and size distribution of tropical fish in temperate waters during expansion.

Fish market surveys are a fundamental approach for understanding the population dynamics of fish species inhabiting surveyed waters, providing a key scientific basis for the sustainable management of fishery resources, such as the diversity and local characteristics of fish species, abundance, biomass, and revenue of fish, and their temporal variability [12–17]. Therefore, long-term monitoring by market surveys may allow us to identify the shifts in fish species compositions and the newly settled species as a response to climate change, and to promote a better understanding of how the newly settled species expand their population in the environment where they made a new entry.

Species belonging to Epinephelidae represent a large portion of catches in tropical and subtropical multispecies fisheries [18]. The Epinephelidae family consists of 16 genera and 163 species, with the *Epinephelus* genus being the most abundant, accounting for approximately half of these species (86 known species) [19]. Although distributed worldwide, they occur primarily in the tropics and subtropics and are economically valuable families that support many large fisheries [18]. Groupers have the characteristics of longevity, late sexual maturation, and aggregation spawning behavior [20], and many groupers are highly prized and prone to overfishing; therefore, some species are cited in the IUCN Red List [18]. Despite the commercial importance of understanding the impact of climate change on grouper species, little is known about how groupers expand their distribution range due to ocean warming.

In this study, we demonstrate changes in the abundance of seven grouper species, including temperate and tropical species, that landed at the Fukue Fish Market on the Goto Islands, Japan, based on a daily market survey conducted from 2008 to 2013. This study aimed to clarify the change in the catch number and size of groupers and to determine the differences in these characteristics between temperate (*Epinephelus awoara*, *E*. *akaara*, and *Hyporthodus septemfasciatus*, *E*. *bruneus*), and tropical groupers (*E*. *fasciatus*, *E*. *areolatus*, and *Plectropomus leopardus*). Next, we discuss the potential factors affecting these transitions in terms of ecological traits and the effects of ocean warming.

## Materials and methods

### Study location and fish market survey

**Fig 1:**
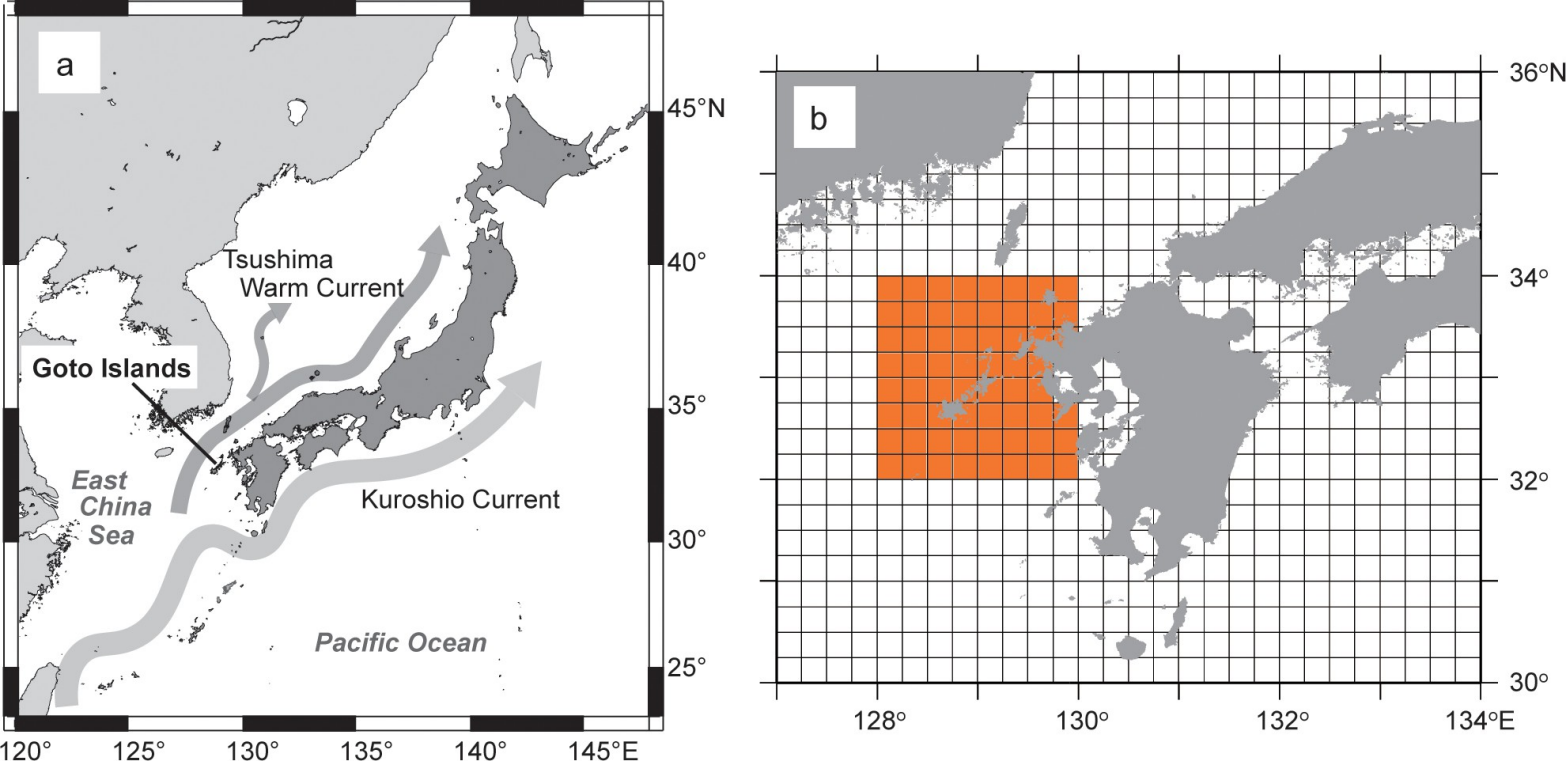
Map of the Goto Islands located in the northwestern part of Kyushu district, Japan. (a) Location of the Goto Islands and major currents affecting the coastal environment around the Goto Islands. (b) The analyzed area (orange color) of seasonal mean SST ranged from 31° N128° E to 34° N 130° E. The area has 94 grid boxes where the MGDSST exist. These figures were created by using the open-source software GMT 6 (https://www.generic-mapping-tools.org/).

This study was conducted at Fukue Fish Market Ltd., in the Goto Islands of western Japan (Fig 1). Fish at the market were mainly caught in the water surrounding the Goto Islands. The Goto Islands face the East China Sea, and the ocean environment is strongly affected by the Tsushima Warm Current and Kuroshio Current (Fig 1A).

We conducted daily fish measurements of groupers caught and landed between January 2008 and December 2013, except on days when the market was closed [21]. Thus, the cumulative number of survey days during the study period was approximately 320. The fish were caught using different catching gears, such as pole-and-line fishing, spearfishing, long lines, gill nets, and set nets, around the waters adjacent to the Goto Islands. The total length (TL) and body weight (BW) of each fish were measured at a fish market. All fish landed with complete bodies without gutting. Moreover, we recorded the fishing method and name of the fishermen/fishing vessel for each fish when available. Thus, the sum of the annual and monthly total number of days each fisherman or fishing vessel landed groupers among all fishermen and fishing vessels was calculated as an index of annual or monthly fishing effort. The nominal catch per unit effort (CPUE) was calculated for each grouper species caught using each fishing method. The nominal CPUE was defined as the total annual or monthly catch number of fish caught by each fishing method divided by the fishing effort index (the sum of the total number of days when each fisherman/fishing vessel landed groupers). As our study only measured the fish length and weight of fish landed in the market, no concerns related to animal ethics were raised. All data from our measurement surveys are available in S1 Table.

### Species identification and classification of groupers

The present study focused on seven grouper species: the yellow grouper (*Epinephelus awoara*), redspotted grouper (*E*. *akaara*), sevenband grouper (*Hyporthodus*. *septemfasciatus*), longtooth grouper (*E*. *bruneus*), blacktip grouper (*E*. *fasciatus*), areolate grouper (*E*. *areolatus*), and leopard coral grouper (*Plectropomus leopardus*). Species identification and nomenclature followed Senou [22], and Craig and Hasting [23]. We classified the seven grouper species into two categories according to the list of FAO major fishing areas following Heemsra and Randall [20]: temperate groupers: *E*. *awoara*, *E*. *akaara*, and *H*. *septemfasciatus*, *E*. *bruneus*; and tropical groupers: *E*. *fasciatus*, *E*. *areolatus*, and *P*. *leopardus* (S2 Table). On the basis of the list by Heemsra and Randall [20], the species that the major fishing area is only temperate regions (Area 61: Pacific, Northwest) was regarded as a temperate grouper in this study (S2 Table). Species that were widely distributed in several major fishing areas including tropical regions (Area 71: Pacific, Western Central), were regarded as tropical grouper (S2 Table).

### Sea surface temperature data

To discuss the relationship between sea surface temperature (SST) and catch trends of groupers, we used merged satellite and in-situ global daily sea surface temperature (MGDSST) data from the Japan Meteorological Agency between 1986 and 2013. The MGDSST data were gridded daily at a spatial resolution of 0.25° × 0.25° [24]. We calculated the quarterly mean SST averaged over the area off the west coast of Kyushu, ranging from 31° N to 34° N and 128° E to 130° E (94 square grids, Fig 1B). To understand the long-term SST trend, we calculated the SST anomalies from the mean values from 1986 to 2013.

## Results

The total length (TL) and body weight (BW) of the groupers were measured for 100,491 individuals, including 29,012 *Epinephelus awoara*, 5,425 *E*. *akaara*, 5,687 *Hyporthodus septemfasciatus*, 5,332 *E*. *bruneus*, 45,181 *E*. *fasciatus*, 6,475 *E*. *areolatus*, and 3,379 *Plectropomus leopardus* in the Fukue Fish Market during the study period (Table 1).

**Table 1:** Total lengths and body weights of each grouper landed at the Fukue Fish Market, Japan between 2008 and 2013.

Classification	Species	*N*	TL (cm)		BW (g)	
			Mean ± S.D.	Min.–Max	Mean ± S.D.	Min.–Max
**Temperate grouper**	***E*. *awoara***	29,012	36.9 ± 5.4	17–62	847.1 ± 378.0	70–3,100
	***E*. *akaara***	5,425	40.0 ± 5.9	20–57	1,079.8 ± 508.8	100–4,300
	***H*. *septemfasciatus***	5,687	47.5 ± 10.4	17–121	1,920.8 ± 1,470.1	100–25,400
	***E*. *bruneus***	5,332	57.6 ± 16.8	18–129	3,945.9 ± 3,791.1	100–35,000
**Tropical grouper**	***E*. *fasciatus***	45,181	30.0 ± 5.5	13–53	470.1 ± 285.8	50–2,800
	***E*. *areolatus***	6,475	41.8 ± 6.2	20–57	1,128.6 ± 489.4	100–3,200
	***P*. *leopardus***	3,379	57.1 ± 12.4	18–90	3,262.9 ± 2,044.5	100–10,800

TL: total length, BW: body weight

Fishing methods were identified in 99,892 individuals (99.9% of the measured groupers). The groupers were mostly caught using the pole-and-line fishing method (79% of all species, S1 Fig). *E*. *awoara* was caught specifically using trap pots (15.3%),and *P*. *leopardus* by spearfishing (31.1%, S1 Fig). Moreover, 58.8% of the *E*. *akaara* and 26.2% of the *E*. *bruneus* were caught using gill nets (S1 Fig). The names of fishermen/fishing vessels were identified for 99,931 fish (99.4%).

### Annual changes in catch trends

**Fig 2:**
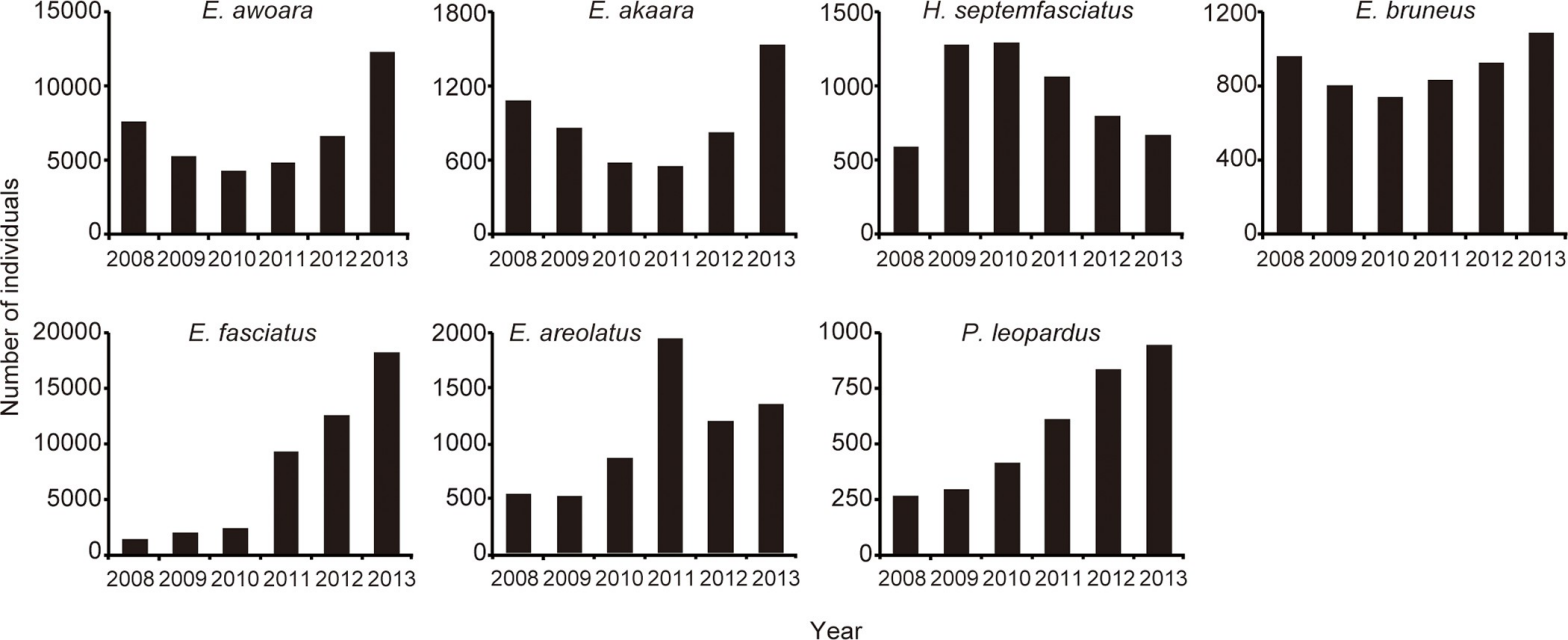
Changes in the annual catch number of seven species of groupers landed at the Fukue Fish Market on the Goto Islands, Japan during the study period (2008–2013).

The annual catch number of fish from 2008 to 2013 showed different trends between temperate and tropical species. The catch trends of temperate species did not exhibit clear changes, although there were some fluctuations throughout the survey period, whereas those of all tropical species increased rapidly, particularly in 2011 (Fig 2). The total catch numbers of the four temperate species, and three tropical species exhibited 1.39-, and 5.9-fold increases on average over six years, respectively.

**Fig 3:**
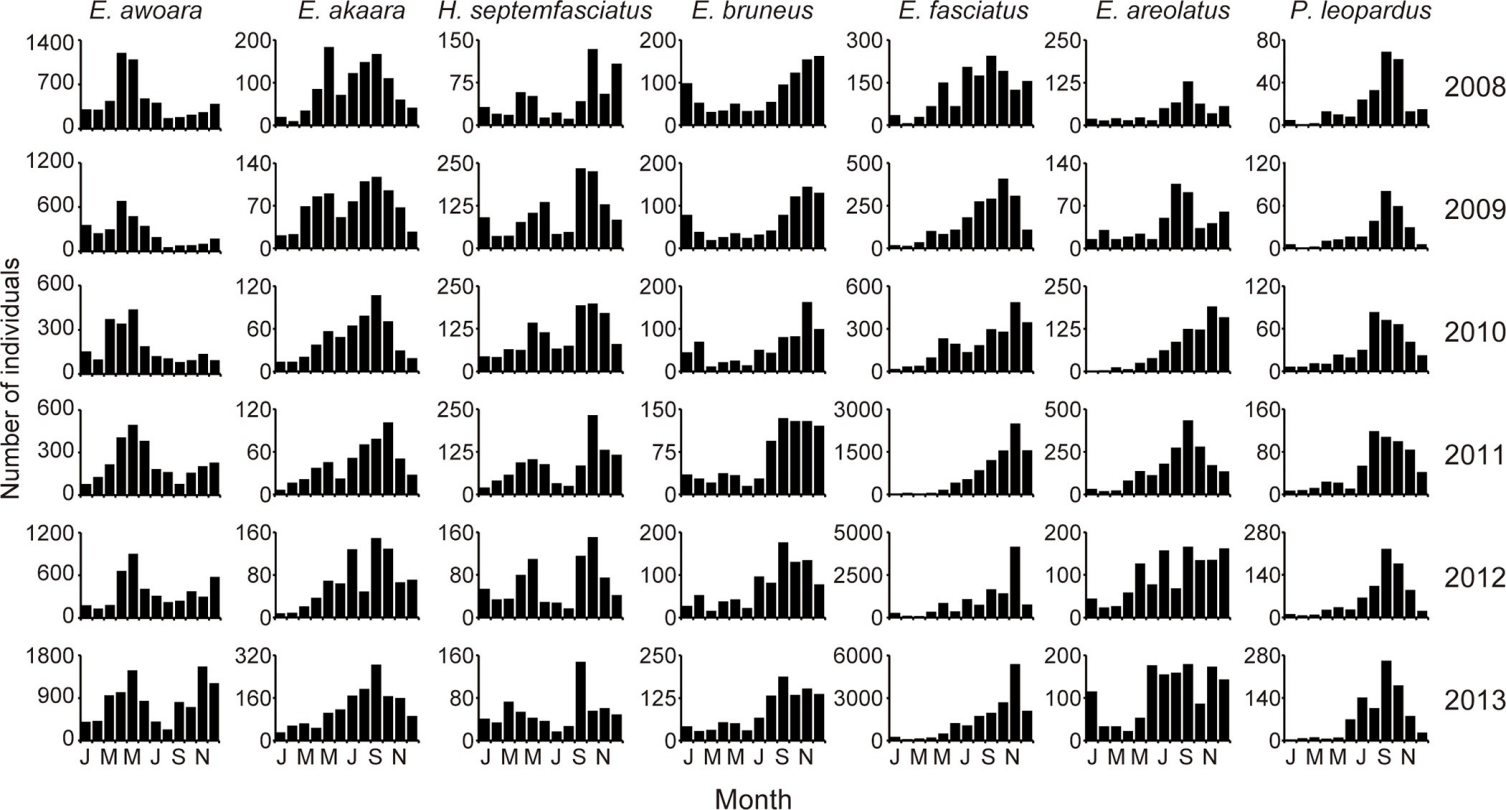
Histograms of the monthly catch number of seven species of groupers landed at the Fukue Fish Market on the Goto Islands, Japan throughout the study period (2008–2013).

The monthly catch numbers of fish throughout the study period showed that all species of tropical grouper and *E*. *bruneus* had peak catch numbers from July to December (Fig 3). However, these trends remained unchanged during the study period. Temperate groupers except for *E*. *bruneus* had bimodal peaks in monthly catch numbers between April and June and between September and November in most years (Fig 3). For *E*. *awoara*, the peak between September and November became more pronounced as the years progressed. *E*. *akaara* had bimodal peaks in the histogram of monthly catch numbers, but gradually lost its peak during April–June and finally exhibited a single peak during September–October 2013. *H*. *septemfasciatus* exhibits bimodal peaks throughout the study period.

### The size distributions

**Fig 4:**
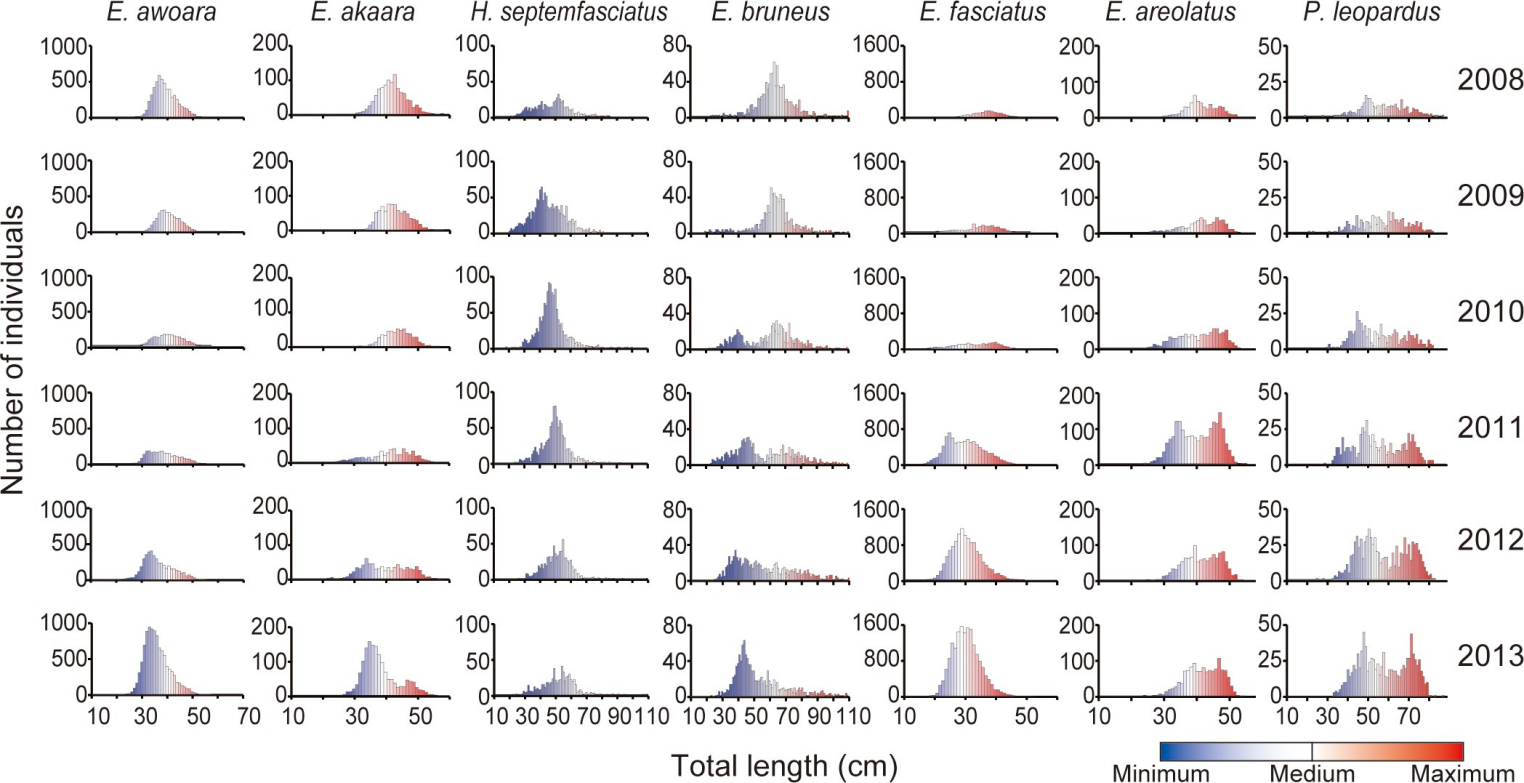
Size distribution of seven species of groupers landed at the Fukue Fish Market on the Goto Islands, Japan throughout the study period (2008–2013). The colors of the bar graphs indicate the relative size of each species.

The size distribution and transition of each species during the study period are shown in Fig 4. *E*. *awoara* and *H*. *septemfasciatus* exhibited almost normal distribution curves throughout the study period, whereas a few size-based cohorts were observed in the histograms of the remaining five species. For *E*. *akaara* and *E*. *bruneus*, the larger-sized cohort gradually disappeared,; however, the smaller-sized cohort became more pronounced as the years progressed. Larger-sized cohorts of the three tropical grouper species were caught early during the study period. They exhibited a drastic increase in the number of individuals in their smaller-sized cohorts (23–26 cm TL for *E*. *fasciatus*, 34–35 cm TL for *E*. *areolatus* and 37–45 cm TL for *P*. *leopardus*) during 2010 and 2011. Thereafter, this smaller-sized population continued to expand.

The mean TL significantly decreased over the six-year study period for *E*. *awoara* (One-way ANOVA, F1,5 = 8.8, P < 0.05), *E*. *bruneus* (F1,5 = 101.4, P < 0.01), and *E*. *fasciatus* (One-way ANOVA, F1,5 = 23.1, P < 0.01). In contrast, *H*. *septemfasciatus* showed a significant but gradual increase in mean TL over the study period (F1,5 = 14.1, P < 0.01). There were no significant trends in TL across the years in *E*. *akaara* (F1,5 = 6.6, P = 0.06), *E*. *areolatus* (F1,5 = 0.1, P = 0.82), or *P*. *leopardus* (F1,5 = 0.1, P = 0.76).

The total length of the caught fish varied significantly among fishing methods for all seven species (S3 Table). Fish caught in trap cages and trap pots tended to be smaller for all grouper species, except for *E*. *fasciatus* (S2 Fig).

### Quarterly changes in sea surface temperature

**Fig 5:**
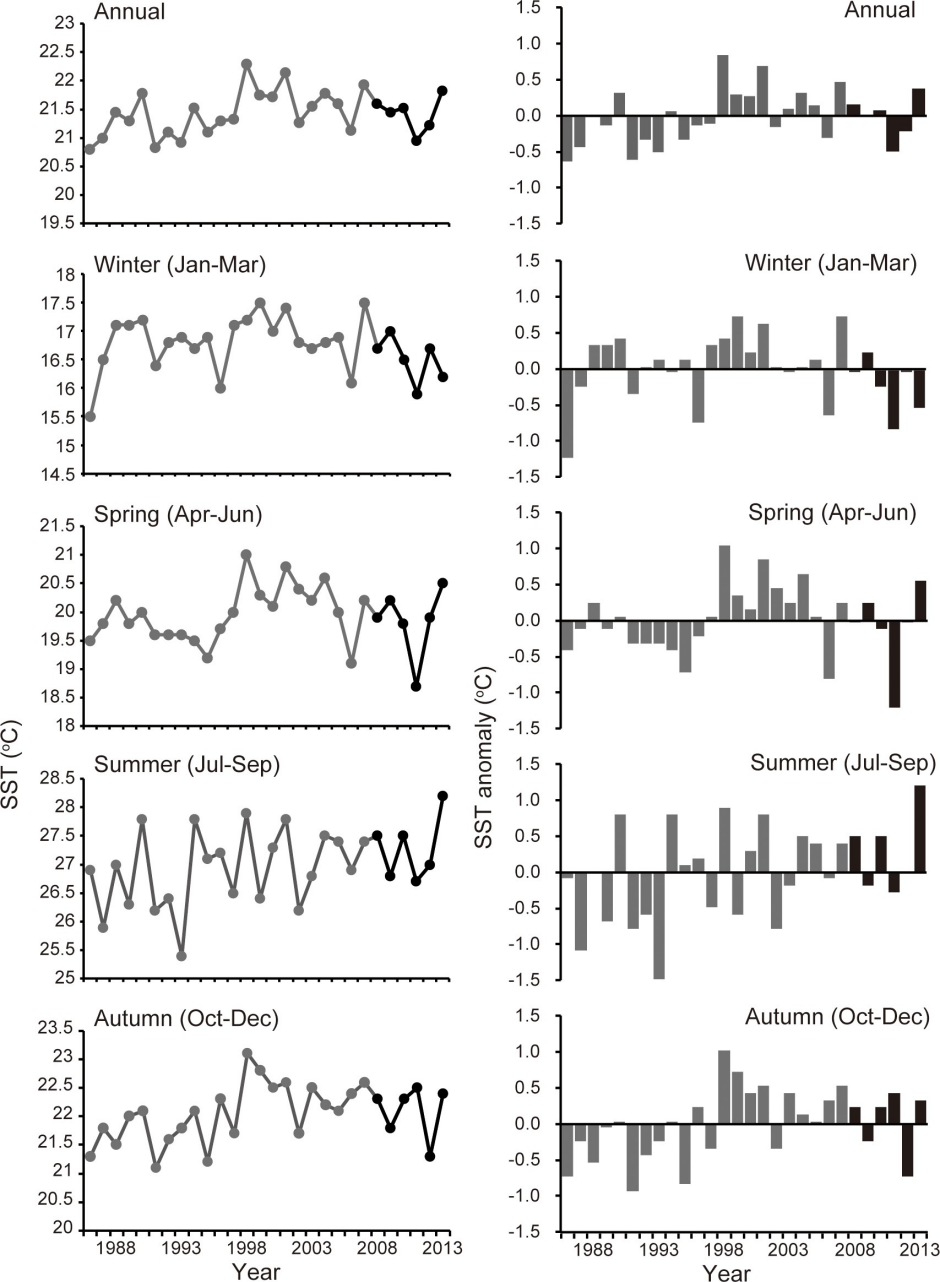
Annual mean sea surface temperature (SST) (*left*) and SST anomaly from the average value (*right*) in the area of the west coast of Kyushu and those in four quarterly seasons from 1986 to 2013. Black bars represent the SST anomaly during our research period, whereas gray bars represent the data before our research. The area of the west coast off Kyushu ranged from 31° N to 34° N, and from 128° E to 130° E.

Mean SSTs in four quarters of the season from 1986 to 2013 were 16.8 ± 0.5°C during the winter (January–March), 19.9 ± 0.5°C during the spring (April–June), 27.0 ± 0.7°C during the summer (July–September), and 22.1 ± 0.5°C during the autumn (October–December), respectively (Fig 5). The annual SST in the waters adjacent to the Goto Islands showed an increasing trend during 1986 to 2013, but it was not significant (F1,26 = 3.1, P = 0.09). The annual SST was relatively lower from 1986 to 1997 but became warmer between 1998 and 2005 (Fig 5). Since 2006, SST has fluctuated and has not shown a clear trend. The SST showed different trends among the four seasons from 1986 to 2013. Winter had a relatively warmer period from 1997 to 2001 (Fig 5), but it fluctuated throughout the period from 1986 to 2013 and did not show a significant trend (F1,26 = 0.06, P = 0.81). The maximum of winter SST was recorded in 2007, just a year before our survey (Fig 5). In spring, the SST experienced a warmer period from 1998 to 2004, whereas relatively lower temperatures were recorded during other periods (Fig 5). Thus, it did not show a significant trend (F1,26 = 0.47, P = 0.51). From summer to autumn, the SST has often recorded temperatures higher than the mean values since 1998 (Fig 5). A significant increasing trend was observed between 1986 and 2013 (summer: F1,26 = 4.49, P < 0.05; autumn: F1,26 = 5.21, P < 0.05).

## Discussion

We conducted daily measurement surveys of groupers landing around the temperate waters of the Goto Islands over six years (2008–2013), demonstrating that the catch number and size of each grouper changed substantially across months and years. In particular, tropical groupers exhibited a remarkable increase in population around the Goto Islands. Prior to the present study, there were no statistical catch data for grouper species in the regions around the Goto Islands, including the Fukue Fish Market. Thus, it was not possible to compare the catch trends of grouper species between the study period (2008–2013) and the years before the 2000s. However, a comparison of the distributions of groupers described in several published studies [22, 25–28] (S4 Table) indicates that these tropical species were first identified in the waters around the Goto Islands between 2000 and 2008. Therefore, the populations of the three tropical grouper species substantially increased in the temperate waters of the Goto Islands from 2008 to 2013.

Tropical groupers increased their population 5.9-fold on average in temperate waters within six years. Surprisingly, *E*. *fasciatus* exhibited a 12.3-fold increase in population size, which may be explained by the fact that the shift rate is faster in fish with smaller body sizes and shorter life cycles [11]. To the best of our knowledge, such rapid increments in catch numbers have never been reported, although there have been a few reports on the extent of population increments of tropical fish in temperate waters (e.g., [29]). Large-scale ocean currents are likely to play a central role in their transport during the larval period in tropical waters [3, 10]. The Goto Islands face the Tsushima Warm Current branching from the Kuroshio Current (Fig 1), which may facilitate larval transport from tropical waters and consequently accelerate shifting and population growth rates on the Goto Islands. A previous numerical simulation study [30] reported that particles assumed to be fish larvae were transported northward (toward the Goto Islands) from the origin of the Tsushima Warm Current from 2006 to 2017, including our research period, compared to the period from 2000 to 2005. This increased transport is attributed to the prominent northward current around the origin of the Tsushima Warm Current [30]. The strengthening of the Tsushima Warm Current may enhance the possibility of grouper larvae settling as they are transported from more distant spawning sites.

Patterns of larval survival and dispersal play key roles in the dynamics of reef fish populations and the expansion of their distribution range (establishment of new habitats) [5, 31–33], although growing evidence has shown that larval retention near spawning sites is more common than previously thought [34–36]. The distance of larval transport in groupers ranges from 30 to 850 km (reviewed in [33]). Therefore, the most reasonable hypothesis for the increase in tropical grouper populations is that tropical grouper larvae transported from tropical waters successfully survive, settle, and overwinter in temperate waters [5, 8, 31]. Thus, the rapid increase in tropical groupers in temperate waters may have been caused by water temperature a few years before it occurred. In 2011, two species of tropical grouper drastically increased their populations in size cohorts of 23–26 cm TL for *E*. *fasciatus* and 34–36 cm TL for *E*. *areolatus*, which corresponded to 2–3 and 4 years, respectively, based on the length-age relationship [37, 38]. For the remaining tropical grouper species, *P*. *leopardus*, moreover, the prominent cohort appeared in a size class of approximately 45 cm TL in 2010, corresponding to an age of 5 years [39]. These facts imply that the water temperature from 2005 to 2009 may have had a critical effect on the survival of fish larvae and juveniles, and the settlement of this species.

Winter temperatures in temperate waters are a key bottleneck for the survival and population establishment of tropical fishes [6, 8]. Although winter SSTs were relatively low during our survey period (2008–2013) over a quarter-century, the highest winter SST between 1986 and 2013 was recorded in 2007, which was only a year prior to our research. Therefore, the warmest winter SST in 2007 might have enhanced the survival rate of tropical grouper juveniles transported from tropical waters, and consequently their settlement rate in the temperate waters of the Goto Islands.

Moreover, relatively higher SSTs were recorded in summer and autumn from 2004 to 2009. The spawning seasons of the three species of tropical groupers around the waters of the Goto Islands are from June to August for *E*. *fasciatus* and *P*. *leopardus* [39, 40], and from July to September for *E*. *areolatus* [38]. Therefore, higher temperatures during early life stages (egg to juvenile) might enhance survival and growth rates, and the larger body size of juveniles may reduce predation risk in temperate waters, even in winter [6].

Another hypothesis is that mature adult fish migrate from southern tropical waters to the Goto Islands, settle, and reproduce there; consequently, their offspring increase their population. This is supported by our results, which showed that all three species of tropical grouper first increased their population in larger size classes, and then increased in smaller classes. Of the three tropical grouper species, migration behavior has only been reported for *P*. *leopardus*, with the farthest distance of migration being less than 20 km [41–43]. Moreover, to the best of our knowledge, there is no empirical evidence that adult migration induces population increments in temperate waters and extends their distribution poleward, although temporal migration in some species of coastal fish, including the grouper (*Epinephelus lanceolatus*) has been reported (e.g., [9]). These facts do not support the hypothesis of adult migration to temperate waters but do not deny its possibility. Therefore, further studies are needed to assess the migration behavior of mature adult fish from tropical groupers.

In addition to the possibility of the new recruitment of tropical groupers from tropical regions to the temperate Goto Islands, the rapid increase in their populations since 2011 may have been caused by self-recruitment from populations that settled on the Goto Islands. Among the tropical groupers, smaller cohorts appeared in 2011 (23–26 cm TL for *E*. *fasciatus*, 34–36 cm for *E*. *areolatus*, and 37–38 cm for *P*. *leopardus*), corresponding to 2–3 years of age for all three species [37–39]. Therefore, it is possible that the rapid increase in 2011 was caused by the offspring of the populations that settled between 2008–2009 and before.

Fishing effort should also be considered as a potential factor affecting increases in the catch of tropical grouper species. *E*. *fasciatus* and *E*. *areolatus* were mainly caught by pole-and-line fishing, whereas *P*. *leopardus* was caught by both pole-and-line fishing and spearfishing. The number of fish caught using both methods increased sharply after 2011 (S1 Fig). In addition, the nominal CPUE for the three tropical grouper species caught using these two main fishing methods increased in synchrony with the catch number (S3 Fig). These results indicate that the populations of the three tropical grouper species have increased in the temperate Goto Islands.

The four species of temperate grouper did not show significant trends of increase/decrease in the number of fish caught but showed some fluctuations during the study period. These species were mainly caught using pole-and-line fishing, as is the case for tropical groupers, although *E*. *akaara* and *E*. *bruneus* were also caught using gill nets (S1 Fig). The nominal CPUE obtained using these fishing methods for each temperate species did not show substantial changes (S3 Fig), indicating that the populations of these groupers did not change, and that the annual fluctuations observed in catch numbers were presumably due to fishing efforts.

The size distributions of *E*. *awoara*, *E*. *akaara*, and *E*. *bruneus* indicated that the larger cohorts decreased over six years, and the smaller size increased. These shifts in dominant size would cause miniaturization of the mean size of these species. Overfishing causes a significant decrease in the mean length of groupers (e.g., [44]). Therefore, these three species may have been over-fished during the study period. *H*. *septemfasciatus* gradually increased in size, indicating that fishing for this species occurred under sustainable conditions. Because *H*. *septemfasciatus* is distributed in relatively deeper waters than the other six species in this study (S2 Table), the difficulty of catching this species may explain the moderate fishing pressure.

For *E*. *akaara*, peaks in catch number were observed around May, but this peak gradually disappeared as the years progressed. This peak corresponds to the spawning season adjacent to the waters around the Goto Islands [45]. This decrease in catch numbers during the spawning season was associated with a decrease in the number of larger individuals during 2008–2010, which played a central role in the breeding populations. Many grouper species form spawning aggregations during their reproductive period [46, 47], although this has not been reported for *E*. *akaara*. Thus, the decrease in catch number and fish size of *E*. *akaara* may have been caused by the overharvesting of mature fish that formed spawning aggregations.

In conclusion, our daily market survey demonstrated that tropical groupers substantially increased in population in temperate waters within only six years, and the population transition dynamics during the expansion process. Although larval transport from tropical waters is still the most likely hypothesis for the settlement of tropical groupers in temperate waters based on several previous studies [7, 10], our data regarding the transition of size distribution suggest the possible hypothesis that adults migrating from tropical waters first settle in temperate waters and then enhance their populations by reproduction in temperate waters. To test these hypotheses, further studies using novel approaches, such as genetic parentage analysis (e.g., [35]) and bio-logging tracking (e.g., [48, 49]), are needed.

## Supporting information

S1 Fig Changes in the number of fish caught by fishing methods for each species of groupers during the study period (2008–2013). (JPG)

S2 Fig Comparisons of the total length of the fish caught by the fishing methods. GN: gill net, LL: longline, PL: pole-and-line, SN: set net, S: spearfishing, TC: trap cage, TP: trap pot. The numbers written in blue indicate the sample sizes for each fishing method.
(JPG)

S3 Fig Changes in the nominal catch per unit effort of seven species of groupers caught by major fishing methods during the study period (2008–2013). (JPG)

S1 Table All measurement data of seven species of groupers landed at the Fukue Fish market on the Goto Islands, Japan during 2008–2013. (XLSX)

S2 Table Biogeographic regions, habitats and reproductive information of seven grouper species. (DOCX)

S3 Table Statistical results of one-way ANOVA for investigating the differences in total length of groupers caught between various fishing methods. (DOCX)

S4 Table The presence of descriptions indicating grouper inhabitation in the waters around the Goto Islands in published literature. (DOCX)
