# Supplementary figures and images for: A rapid increase in tropical species of grouper (Perciformes: Serranidae) in the temperate waters, the Goto Islands, Japan
Source: PLoS One. 2024 Sep 18;19(9):e0308715. doi: 10.1371/journal.pone.0308715 (PMC11410230; doi:10.1371/journal.pone.0308715)

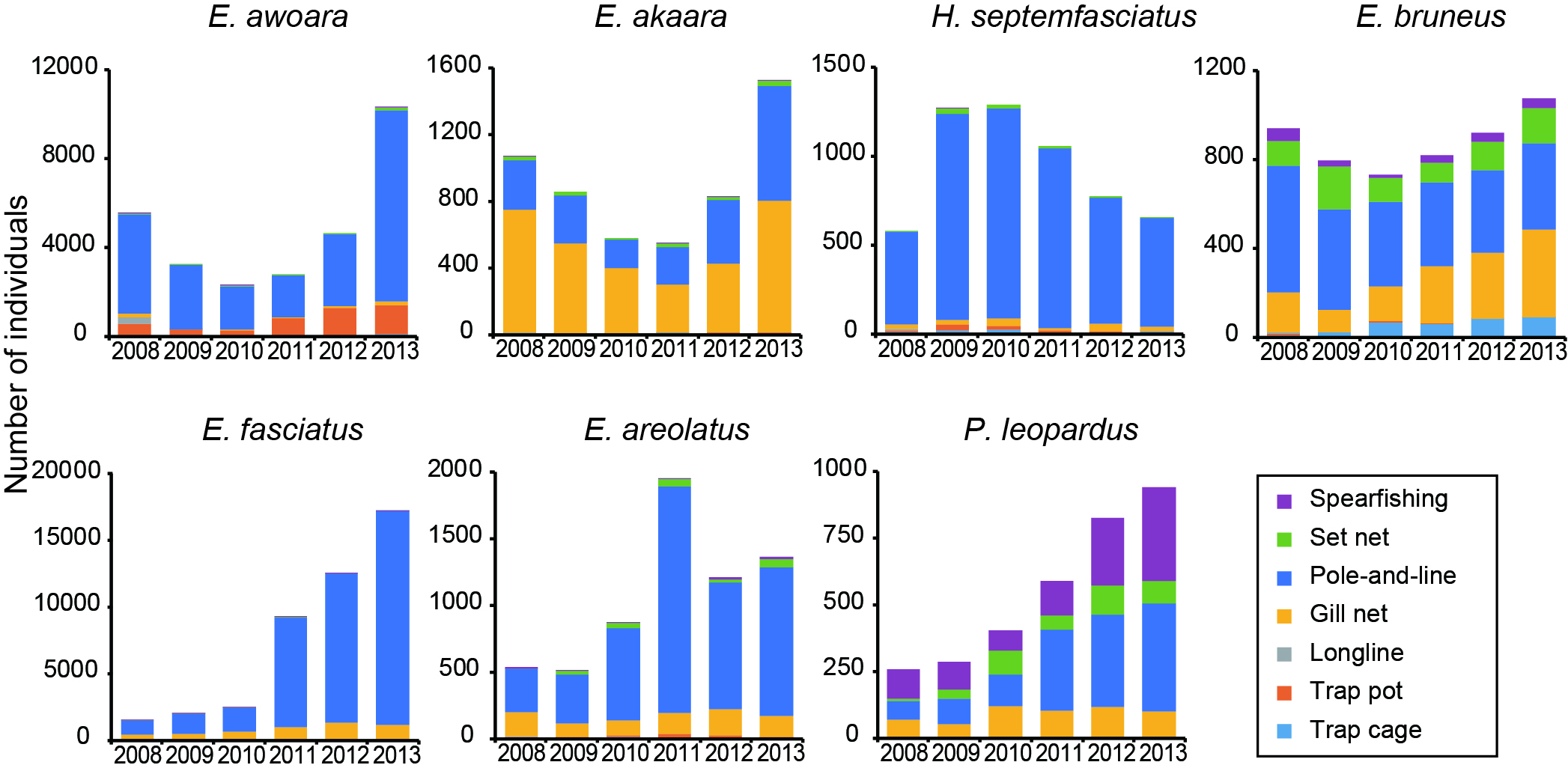

Supplement: S1 Fig — (JPG) [file pone.0308715.s001.jpg]

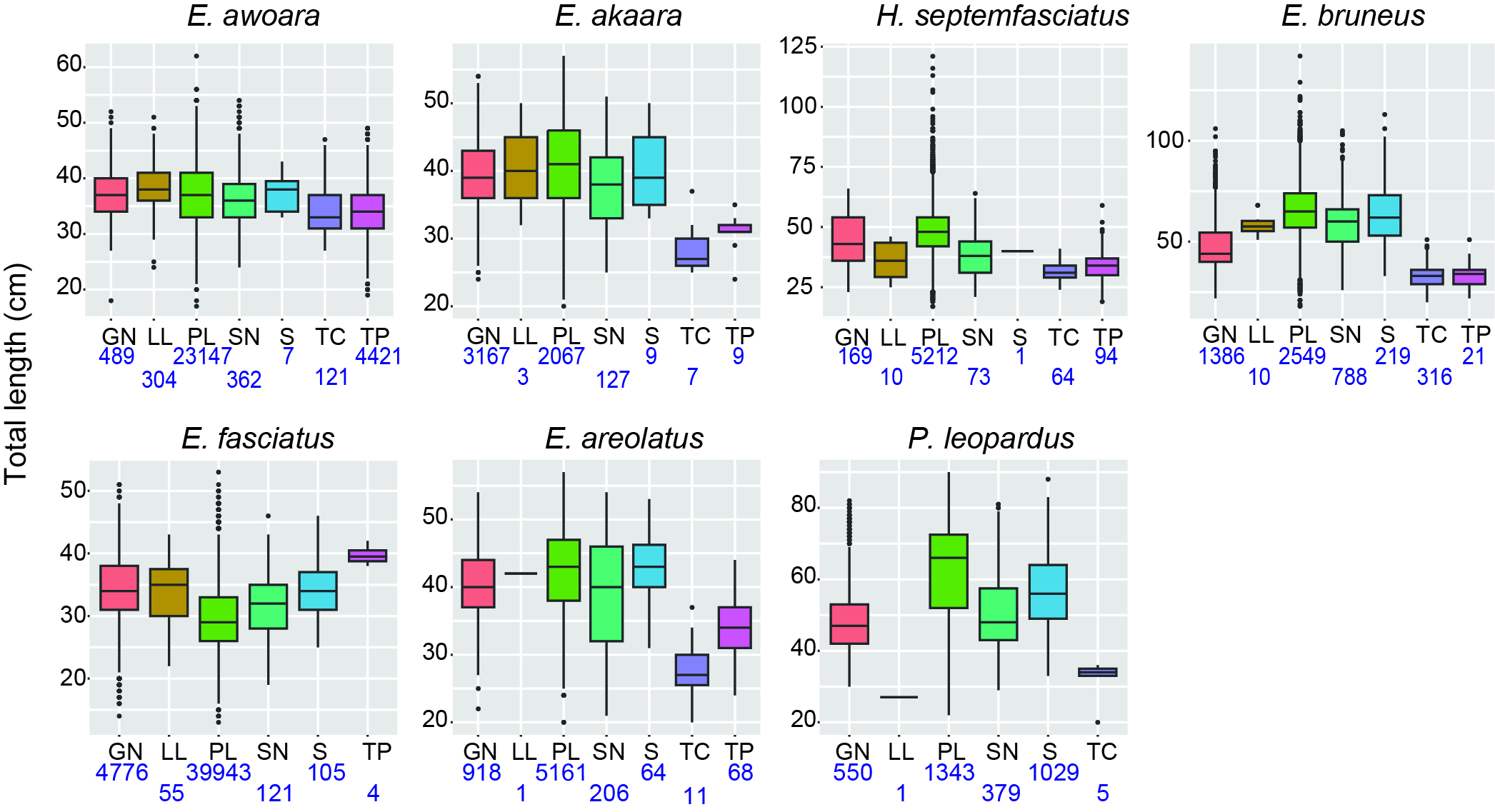

Supplement: S2 Fig — GN: gill net, LL: longline, PL: pole-and-line, SN: set net, S: spearfishing, TC: trap cage, TP: trap pot. The numbers written in blue indicate the sample sizes for each fishing method. (JPG) [file pone.0308715.s002.jpg]

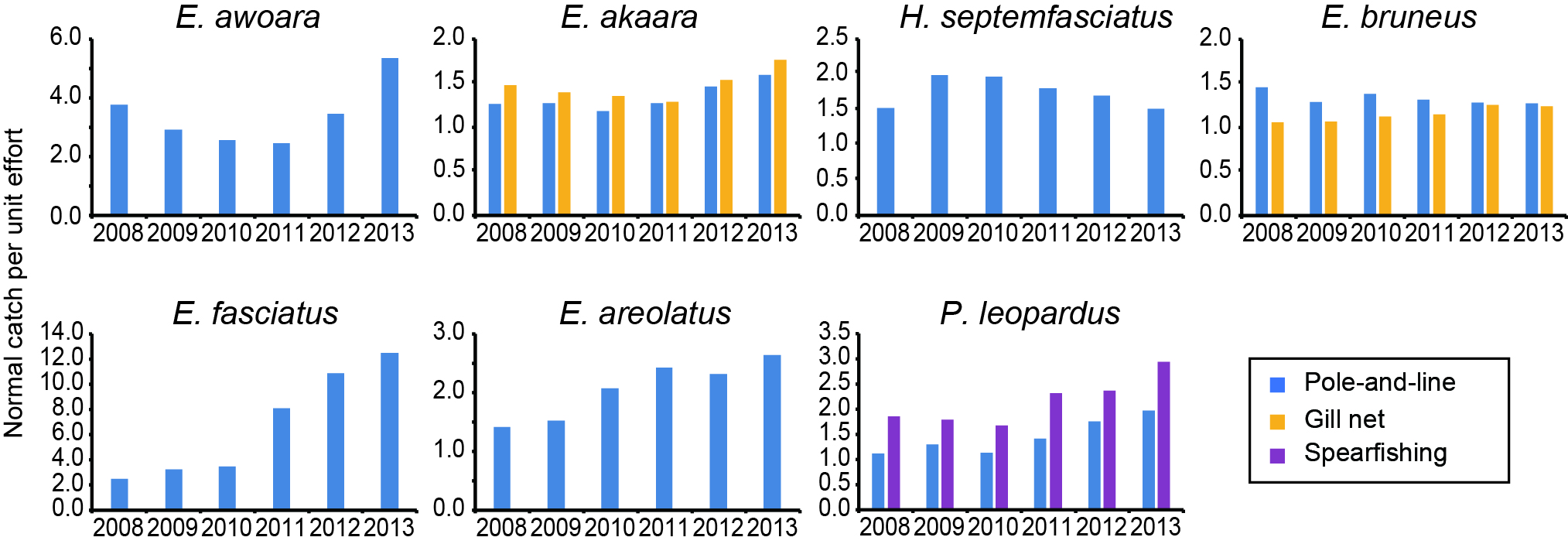

Supplement: S3 Fig — (JPG) [file pone.0308715.s003.jpg]
