# Supplementary material for: A rapid increase in tropical species of grouper (Perciformes: Serranidae) in the temperate waters, the Goto Islands, Japan
Source: PLoS One. 2024 Sep 18;19(9):e0308715. doi: 10.1371/journal.pone.0308715 (PMC11410230; doi:10.1371/journal.pone.0308715)
Supplement: S2 Table — (DOCX) [file pone.0308715.s005.docx]

**Table S2.** Biogeographic regions, habitats and reproductive information of seven grouper species.

| Distribution | Species | FAO Major fishing area [1] | Habitat [1–4] | Depth range [1–4] | Spawning season | Age of first maturity as a female) |
| --- | --- | --- | --- | --- | --- | --- |
| Temperate | *E. awoara* | Northwest Pacific | Rocky reefs | 3–50 m | May–July [5] | >2 [5] |
|  | *E. akaara* | Northwest Pacific | Rocky reefs | 5–55 m | June–August [6] | 3–6 [6] |
|  | *H. septemfasciatus* | Northwest Pacific | Rocky reefs | 4–300 m | June–July [7] | Unknown |
|  | *E. bruneus* | Northwest Pacific* | Rocky reefs | 1–200 m | May–July [8] | 5–9 [12] |
| Tropical | *E. fasciatus* | Southwest Atlantic, Western Indian Ocean, Eastern Indian Ocean, Northwest Pacific, Western Central Pacific, Eastern Central Pacific, Southwest Pacific | Rocky and coral reefs | 2–160 m | June–August [9] | 2–3 [9] |
|  | *E. areolatus* | Western Indian Ocean, Eastern Indian Ocean, Northwest Pacific, Western Central Pacific | Rocky and coral reefs | 2–200 m | June–August [10] | 2–4 [10] |
|  | *P. leopardus* | Eastern Indian Ocean, Northwest Pacific, Western Central Pacific | Rocky and coral reefs | 3–100 m | June–August [11] | 4–5 [11] |
| * Major fishing area for this species was assigned to Areas 61 (Northwest Pacific),and 71 (Western Central Pacific) in the list of Heemstra and Randall 1993 [1]. In the description for each species, however, there is no description that *E. bruneus* inhabit or is caught in the regions of Area 71. Therefore, this study classified the major fishing area of *E. bruneus* is only Area 61. | | | | | | |

**References**

1. Heemstra PC, Randall JE. Groupers of the world. FAO Species Catalog 16. Roma; FAO; 1993.
2. Craig MT, Sadovy de Mitcheson Y, Heemstra PC. Groupers of the World: a Field and Market Guide. NISC Ltd; 2011.
3. Senou H. Family Serranidae. In: Nakabo T. editor. Fishes of Japan with Pictorial Keys to the Species 3rd Edition, Kanagawa, Tokai University Press; 2013. pp757–802.
4. Ikeda H, Nakabo T. Fishes of the Pacific coasts of southern Japan. 1st ed. Kanagawa, Tokai University Press; 2015. (in Japanese)
5. To WL. The biology, fishery of groupers (Family: Serranidae) in Hong Kong and adjacent waters, and implications for management. Ph.D. Thesis, The Hong Kong University. 2009.
6. Kawano M, Nanbu T. Age, Growth and Maturation of Red spotted grouper *Epinephelus akaara* in Waters off Yamaguchi Prefecture, southwestern Japan Sea. Bull Yamaguchi Pref Fish Res Ctr. 2023; 20:1−8. (in Japanese)
7. Yamada U, Tokimura M, Horikawa H, Nakabo T. Fishes and Fisheries of the East China and Yellow Seas. Kanagawa, Tokai University Press; 2007. (in Japanese)
8. Agari T. Maturity characteristics of female longtooth groupers (*Epinephelus bruneus*) in the waters off Nagasaki Prefecture. Achievements in fisheries research and development. 2019. (in Japanese) (https://fra-seika.fra.go.jp/~dbmngr/cgi-bin/search/search_detail.cgi?RESULT_ID=8363&YEAR=2019)
9. Sakino R. Habitat expansion of blacktip groupers (*Epinephelus fasciatus*) due to ocean warming: Implications from physiological analysis of growth and reproductive status. M.Sc. Thesis, Nagasaki University. 2024. (in Japanese)
10. Kume G, Oyama K, Hikichi K, Moritoshi HE. Life history characteristics of the protogynous hermaphroditic areolate grouper *Epinephelus areolatus* in Kagoshima Bay, southern Japan. Environ Biol Fish. 2023; 106:1357–1369.
11. Okuyama J, Shishidou H, Ebisawa A, Yamaguchi T, Nakagawa M. The impact of global warming on coastal fish species inferred from the latitudinal cline observed in the resource characteristics of the regional populations of coral groupers. Bull Japan Soc Fish Oceanogr. 2023; 87:198–199. (in Japanese)
12. Agari T. A study on resource enhancement of longtooth groupers (*Epinephelus bruneus*). Fish Dev. 2018; 130: 7–12. (in Japanese)
