# Supplementary material for: A rapid increase in tropical species of grouper (Perciformes: Serranidae) in the temperate waters, the Goto Islands, Japan
Source: PLoS One. 2024 Sep 18;19(9):e0308715. doi: 10.1371/journal.pone.0308715 (PMC11410230; doi:10.1371/journal.pone.0308715)
Supplement: S3 Table — (DOCX) [file pone.0308715.s006.docx]

**S3 Table.** Statistical results of one-way ANOVA for investigating the differences in total length of groupers caught between various fishing methods.

|  | df | *F* | *P* |
| --- | --- | --- | --- |
| *E. awoara* | 6, 28844 | 263.17 | <0.001 |
| *E. akaara* | 6, 5379 | 20.5 | <0.001 |
| *H. septemfasciatus* | 6, 5623 | 75.924 | <0.001 |
| *E. bruneus* | 6, 5279 | 453.51 | <0.001 |
| *E. fasciatus* | 6, 44997 | 744.07 | <0.001 |
| *E. areolatus* | 6, 6422 | 54.506 | <0.001 |
| *P. leopardus* | 5, 3301 | 140.36 | <0.001 |
