# Supplementary material for: A rapid increase in tropical species of grouper (Perciformes: Serranidae) in the temperate waters, the Goto Islands, Japan
Source: PLoS One. 2024 Sep 18;19(9):e0308715. doi: 10.1371/journal.pone.0308715 (PMC11410230; doi:10.1371/journal.pone.0308715)
Supplement: S4 Table — (DOCX) [file pone.0308715.s007.docx]

**S4 Table.** The presence of descriptions indicating grouper inhabitation in the waters around the Goto Islands in published literature.

| Classification | Species | Katayama 1988 [1] | Senou 2000 [2] | Senou 2013 [3] | Ikeda & Nakabo 2015 [4] | Kuriiwa 2018 [5] |
| --- | --- | --- | --- | --- | --- | --- |
| Temperate | *E. awoara* | **Yes** | **Yes** | **Yes** | **Yes** | **Yes** |
|  | *E. akaara* | **Yes** | **Yes** | **Yes** | **Yes** | **Yes** |
|  | *H. septemfasciatus* | **Yes** | **Yes** | **Yes** | **Yes** | **Yes** |
|  | *E. bruneus** | **Yes** | **Yes** | **Yes** | **Yes** | **Yes** |
| Tropical | *E. fasciatus* | No | No | **Yes** | **Yes** | No |
|  | *E. areolatus* | No | No | **Yes** | No | *N/A* |
|  | *P. leopardus* | No | No | **Yes** | **Yes** | No |

**References**

1. Katayama M. Family Serranidae. In: Masuda H, Amaoka K, Araga C, Ueno T, Yoshino T, editors. The fishes of the Japanese archipelago. 2nd edition. Tokyo: Tokai University Press; 1988 pp. 123−134.
2. Senou H. Family Serranidae. In: Nakabo T. editor. Fishes of Japan with Pictorial Keys to the Species 2nd Edition, Tokyo, Tokai University Press; 2000. pp. 690–731. (*in Japanese*)
3. Senou H. Family Serranidae. In: Nakabo T. editor. Fishes of Japan with Pictorial Keys to the Species 3rd Edition, Kanagawa, Tokai University Press; 2013. pp757–802.
4. Ikeda H, Nakabo T. Fishes of the Pacific coasts of southern Japan. 1st ed. Kanagawa, Tokai University Press; 2015. (*in Japanese*)
5. Kuriiwa K. Family Serranidae. In: Nakabo T. editor. The natural history of the fishes in Japan. Tokyo, Shogakkan; 2018. pp. 232–243. (*in Japanese*)
